# Specific Human Astrocyte Subtype Revealed by Affinity Purified GFAP^+1^ Antibody; Unpurified Serum Cross-Reacts with Neurofilament-L in Alzheimer

**DOI:** 10.1371/journal.pone.0007663

**Published:** 2009-11-04

**Authors:** Jinte Middeldorp, Simone A. van den Berge, Eleonora Aronica, Dave Speijer, Elly M. Hol

**Affiliations:** 1 Department of Astrocyte Biology & Neurodegeneration, Netherlands Institute for Neuroscience, an Institute of the Royal Netherlands Academy of Arts and Sciences, Amsterdam, The Netherlands; 2 Department of (Neuro)pathology, Academic Medical Center, University of Amsterdam, Amsterdam, The Netherlands; 3 Epilepsy Institute in the Netherlands Foundation (Stichting Epilepsie Instellingen Nederland, SEIN), Heemstede, The Netherlands; 4 Department of Medical Biochemistry, Academic Medical Center, University of Amsterdam, Amsterdam, The Netherlands; Brigham and Women's Hospital/Harvard Medical School, United States of America

## Abstract

The human GFAP splice variants GFAPΔ164 and GFAPΔexon6 both result in a GFAP protein isoform with a unique out-of-frame carboxy-terminus that can be detected by the GFAP^+1^ antibody. We previously reported that GFAP^+1^ was expressed in astrocytes and in degenerating neurons in Alzheimer's disease brains. In this study we aimed at further investigating the neuronal GFAP^+1^ expression and we started by affinity purifying the GFAP^+1^ antibody. The purified antibody resulted in a loss of neuronal GFAP^+1^ signal, although other antibodies directed against the amino- and carboxy-terminus of GFAPα still revealed GFAP-immunopositive neurons, as described before. With an in-depth analysis of a western blot, followed by mass spectrometry we discovered that the previously detected neuronal GFAP^+1^ expression was due to cross-reactivity of the antibody with neurofilament-L (NF-L). This was confirmed by double-label fluorescent immunohistochemistry and western blotting with the unpurified GFAP^+1^ antibody and an antibody against NF-L. Our data imply that NF-L can accumulate in some tangle-like structures in Alzheimer brains. More importantly, the purified GFAP^+1^ antibody clearly revealed a specific subtype of astrocytes in the adult human brain. These large astrocytes are present throughout the brain, e.g., along the subventricular zone, in the hippocampus, in the striatum and in the spinal cord of controls, Alzheimer, and Parkinson patients. The presence of a specific GFAP-isoform suggests a specialized function of these astrocytes.

## Introduction

Glial fibrillary acidic protein (GFAP) belongs to class III of the intermediate filament (IF) proteins and is used as a specific marker for astrocytes. Besides expression in astrocytes, GFAP expression has also been observed in non-CNS cells such as Schwann cells [1], [2], fibroblasts [2] and hepatic stellate cells [3], but also in degenerating hippocampal neurons in AD and Down syndrome patients [4], [5]. This neuronal expression became apparent upon studying two novel GFAP splice variants, GFAPΔexon6 and GFAPΔ164. Translation of these out-of-frame splice variants of GFAPα, the canonical GFAP isoform, results in two proteins with the same frameshifted carboxy (C)-terminus, against which we raised a specific antibody named GFAP^+1^. Immunohistochemistry with this antibody revealed that mainly neurons express GFAP^+1^ and only a few astrocytes, contrasting with a commonly used GFAP antibody that clearly stained many astrocytes and also the tangles, but much weaker. The neuronal expression was also obvious when using polyclonal antibodies of Dako, Sigma and raised by Dahl [5]. These remarkable results initiated us to further investigate neuronal GFAP expression.

Here we report that although additional GFAP antibodies, with their epitope mapping at the C-terminus or amino (N)-terminus of human GFAPα, do stain neuron-like structures, neuronal staining with the GFAP^+1^ antibody disappeared after affinity purification of the antibody. Mass spectrometry revealed that the neuronal staining by the GFAP^+1^ antibody was caused by a cross-reaction with neurofilament-L (NF-L). This study shows that some tangle-like neurons in Alzheimer brains accumulate NF-L. Furthermore, we could identify a subpopulation of astrocytes in the human brain by the GFAP^+1^ antibody, which became apparent upon affinity purification.

## Materials and Methods

### Human Post-Mortem Brain and Spinal Cord Material

Human post-mortem paraffin-embedded and frozen brain material, and frozen spinal cord samples were obtained from the Netherlands Brain Bank (NBB), Amsterdam. Spinal cord filaments were purified as described previously [6]. Frozen spinal cord (S06/9) used for immunostaining was obtained from the Amsterdam Medical Center, Amsterdam. More detailed donor information is presented in table 1. Fetal brain material and that of young control donors was obtained from the department of Neuropathology of the Academic Medical Center in Amsterdam.

**Table 1:** Detailed donor information.

Donor number	Area	Sex	Age	Diagnose	Postmortem delay
NBB 96-058	Hippocampus	Female	75	Alzheimer's disease	03:35
NBB 88-073	Hippocampus	Male	66	Alzheimer's disease	03:15
NBB 93-040	Hippocampus	Male	83	Alzheimer's disease	03:15
NBB 01-125	Hippocampus	Female	77	Alzheimer's disease	08:30
NBB 01-119	Hippocampus	Male	65	Alzheimer's disease	08:50
NBB 99-090	Hippocampus	Female	82	Alzheimer's disease	05:20
NBB 95-102	Hippocampus	Male	53	Nondemented control	10:00
NBB 06-037	Hippocampus	Male	66	Nondemented control	07:45
NBB 00-142	Hippocampus	Female	82	Nondemented control	05:30
NBB 01-016	Hippocampus	Male	77	Nondemented control	08:25
NBB 01-025	Hippocampus	Female	76	Nondemented control	04:05
NBB 05-083	Spinal cord &Hippocampus	Female	85	Nondemented control	05:00
NBB 01-021	Caudate/putamen	Male	82	Nondemented control	07:40
NBB 02-013	Caudate/putamen	Female	80	Parkinson's disease	05:30
NBB 07-006	2 areas of SVZ	Male	75	Alzheimer's disease	05:25
NBB 99-046	Spinal cord	Female	89	Nondemented control	05:10
AMC S06-9	Spinal cord	Female	72	Nondemented control	<10:00
N.A.	Hippocampus	Female	39	Nondemented control	07:30

NBB = Netherlands Brain bank; AMC = Amsterdam Medical Center; SVZ = Subventricular zone; N.A. = Not Available.

### Affinity Purification of GFAP^+1^ Antibody

The GFAP^+1^ antibody [5] was affinity-purified by using CnBr-activated Sepharose 4B beads (GE Healthcare, Fairfield, United States) [7]. First 1 gram of CnBr-activated Sepharose 4B beads was incubated in 3.5 ml 1 mM HCl to let them swell. After that, they were washed twice with acetate buffer (0.1 M sodium acetate, 0.5 M NaCl, pH 4.0). Next, 1 ml of the beads was mixed with 400 µg peptide against which the GFAP^+1^ antibody was raised (EDRGDAGWRG; synthesized by the Netherlands Cancer Institute batch 6EH1) in 5 ml coupling buffer (0.1 M boric acid and 0.5 M NaCl pH 8.3). The peptide was coupled to the beads while mixing head over head for approximately 16 hours at 4°C. Subsequently, the beads were washed three times with coupling buffer and incubated with blocking buffer (1 M Glycine, pH 8) for 2 hours at 4°C while rotating head over head. The beads were then washed twice by alternating ammonium formate buffer (0.1 M ammonium formate, pH 2.7) and Tris buffer (0.1 M Tris, 0.5 M NaCl, pH 8), and once more with ammonium formate buffer. After a final wash with PBS (137 mM NaCl, 2.7 mM KCl, 1.8 mM KH_2_PO_4_, and 4 mM Na_2_HPO_4_, pH 7.4) 1 ml GFAP^+1^ antibody with 1 ml PBS was added to the beads in a column and incubated for 1 hour at room temperature. Flow-through (aspecific fraction) was stored in a tube and the specific antibodies were obtained from the column by rinsing it with 4 ml PBS (5 times) and eluting it with 4 ml 0.1 M ammonium formate (6 times). Each elution fraction was immediately neutralized with 3.5 ml 1 M ammonium and stored at −80°C. Western blotting of the different fractions confirmed that most specific antibodies were present in the 1^st^ elution fraction.

### Immunohistochemistry

Paraffin sections were deparaffinised and incubated in 0.3% H_2_O_2_ in methanol for 20 min, before preincubation with 10% normal horse serum (NHS) in phosphate buffer (PB; 0.05 M Na_2_HPO_4_, 0.05 M NaH_2_PO_4_·H_2_O, pH 7.4) + 0.4% Triton X-100 for 30 min. Next, sections were incubated with primary antibodies GFAP^+1^ (bleeding 150498; 1∶1000 [5]), GFAP^+1^ purified (bleeding 120598; 1∶250), pan-GFAP (Dako; 1∶1000), GFAP C-term (sc-6170, Santa Cruz Biotechnology, Inc.; 1∶200) and GFAP N-term (sc-6171, Santa Cruz Biotechnology, Inc.; 1∶200) overnight in PB + 3% NHS + 0.04% Triton X-100. Then, sections were rinsed with PB three times and incubated with secondary biotinylated antibodies (Vector Laboratories; 1∶400) in PB + 1% NHS + 0.4% Triton X-100 for 1 hour at room temperature upon which they were rinsed three times with PB and incubated with the ABC-complex (1∶800 in PB; Vector Laboratories) for 30 min. Sections were washed again three times before incubation with DAB-solution (Sigma; 0.5 mg/ml) for approximately 10 min. Finally, the sections were washed once in PB, twice in distilled water, dehydrated in a graded ethanol-xylene series and embedded in entellan.

For the doublestaining of pan-GFAP and neurofilament, pan-GFAP (Dako; 1∶1000) and pan-neuronal neurofilament (SMI 311, Covance; 1∶1000) antibodies were mixed and incubated together overnight. The next day, after washing, the sections were incubated with anti-rabbit-HRP (Dako; 1∶500) for 1 hour followed by washing and incubation with DAB intensified by nickel ammonium sulphate to get a more black precipitate. After this, the sections were washed again and incubated with biotinylated anti-mouse (Vector Laboratories; 1∶400) for 1 hour, followed by 30 min incubation with the ABC-complex and DAB as described before.

Fluorescent staining of GFAP^+1^ and NF-L was performed on frozen hippocampus and spinal cord tissue. Sections were fixed in 4% paraformaldehyde before blocking for 1 hour in 10% NHS in PB + 0.4% Triton X-100. Subsequently, the sections were incubated with a polyclonal rabbit antibody against GFAP^+1^ (1∶1000) and a monoclonal mouse antibody against Neurofilament-L (NF-L; MAB1615, Chemicon; 1∶200) diluted in PB + 3% NHS + 0.4% Triton X-100 overnight at room temperature. Following, the sections were washed and incubated with secondary fluorescent antibodies anti-rabbit Alexa Fluor® 488 (Molecular Probes; 1∶1400) and anti-mouse Cy3 (Jackson; 1∶1400) and nuclear stain Hoechst 33258 (BioRad; 1∶1000). After washing, the sections were coverslipped with Mowiol (0.1 M Tris pH 8.5, 25% glycerol, 10% w/v Mowiol 4–88 (Sigma)).

### Western Blot

Spinal cord filament samples (pellet fraction) were diluted 1∶20 with 1x loading buffer (50 mM Tris, 2% SDS, 10% glycerol, 100 mM DTT, 0.005% bromophenol blue). Subsequently, the samples were run on a 7.5% SDS-PAGE gel and blotted semi-dry on nitrocellulose. In one experiment, half of the gel was used for Coomassie staining. Blots were preincubated for 10 min in Supermix (0.0 M Tris, 0.9% NaCl, 0.25% gelatin and 0.5% Triton X-100, pH 7.4) before incubation with primary antibodies GFAP^+1^ unpurified (1∶500), GFAP^+1^ purified (1∶250) and NF-L (1∶1000) in Supermix overnight at 4°C. Next, the blots were washed with TBS-T (TBS; 100 mM Tris-HCl pH 7.4, 150 mM NaCl, with 0.2% tween-20) and incubated with secondary antibody anti-rabbit IRDye800 (Rockland Immunochemicals Inc.; 1∶5000) or anti-mouse Cy5 (Jackson; 1∶2000) in Supermix for one hour at RT. After three washes in TBS, bands were visualized with the Odyssey Infrared Imaging System (LI-COR Biosciences, Lincoln, USA).

### MALDI Analysis

For MALDI analysis, protein-containing gel slices were S-alkylated (slices were incubated sequentially with 10 mg/50 mM NH_4_HCO_3_ ml DTT (Sigma) for 30 minutes at 55°C and 25 mg/50 mM NH_4_HCO_3_ ml iodoacetamide (Sigma) for 30 minutes at RT, digested with trypsin (Roche Molecular Biochemicals, sequencing grade) and extracted according to Shevchenko *et al.* [8]. After drying in a vacuum centrifuge, peptides were dissolved in 1% formic acid and 60% acetonitrile (7 µl). Eluted peptides were mixed 1∶1 (v/v) with a solution containing 52 mM α-cyano-4-hydroxycinnamic acid (Sigma-Aldrich Chemie BV) in 49% ethanol/49% acetonitrile/2% TFA and 1 mM ammonium acetate. Prior to dissolving, the α-cyano-4-hydroxycinnamic acid was washed briefly with chilled acetone. The mixture was spotted on a MALDI target plate and allowed to dry at room temperature. Reflectron matrix-assisted laser desorption ionization time of flight mass spectrometry (MALDI-TOF MS) spectra were acquired on a Micromass M@LDI (Wythenshawe, UK). The resulting peptide spectra were interpreted with Micromass proteinprobe software and analyzed with MASCOT peptide mass fingerprint software and databases (both available at http://www.matrixscience.com).

## Results

### Neuronal-Like GFAP Expression Detected by Distinct GFAP Antibodies

**Figure 1:**
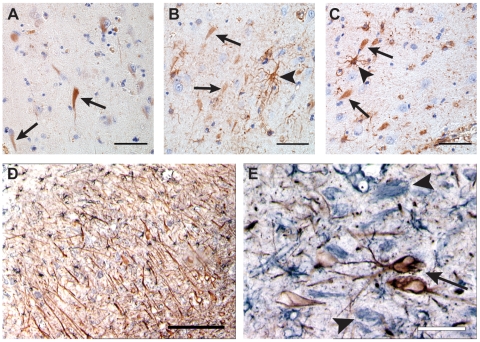
Neuron-like staining by different GFAP antibodies. Neuron-like structures are stained by the GFAP^+1^ (A), GFAP C-terminal (B) and GFAP N-terminal (C) antibody (arrows). The C-terminal and N-terminal antibodies also stain astrocytes (B–C, arrowheads). Double-labeling of pan-GFAP (black) and neurofilament (brown) (D–E) shows that neuron-like structures stained by GFAP (E, arrowheads) are surrounded by neurofilament positive neurons (E, arrow), but do not co-localize. NBB 96-058 (A–C), NBB 88-073 (D–E). Scale bars represent 100 µm (A–C), 200 µm (D), 25 µm (E).

Neuron-like structures were not only stained by the GFAP^+1^ antibody (Fig. 1A, arrows), but also by an antibody against the C-terminus (Fig. 1B, arrows) and N-terminus (Fig. 1C, arrows) of GFAPα. In addition, as expected these last two antibodies also stained the GFAP-IF network of astrocytes in the same area (Fig. 1B–C, arrowheads). Since the C-terminal GFAP antibody should not stain the GFAP^+1^ protein, as the C-terminus of the GFAPΔ164 and GFAPΔexon6 isoforms differ completely from the C-terminus of GFAPα, we concluded that GFAPα is present in the neuronal structures. These data confirm our earlier results with the pan-GFAP antibodies from Dako, Sigma and Dahl [5], which also stained these neuron-like structures (Fig. 1E, arrowheads). However, doublestaining with a pan-neuronal neurofilament antibody (Fig. 1D–E) showed that these GFAP positive neuronal structures (grey) were different from the neurons stained by the neurofilament antibody (brown, Fig. 1E, arrow). Although the neuronal structures were present in the CA1 area of the hippocampus, no co-localization was seen between pan-GFAP and neurofilament. This made us question the specificity of the neuronal GFAP^+1^ staining, which appeared to be different from the pan-GFAP neuron-like staining.

### GFAP^+1^ Antibody Specificity

**Figure 2:**
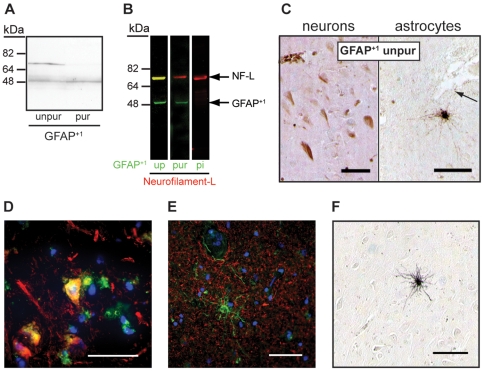
GFAP^+1^ cross-reactivity with neurofilament-L is removed by affinity purification. A Western blot on human spinal cord filaments revealed two bands (∼50 kDa and ∼70 kDa) when stained with the unpurified GFAP^+1^ antibody (A, unpur). After affinity purification of the antibody only the lower band was left (A, pur). On a western blot that was double-stained for different GFAP^+1^ antisera (green) and NF-L (red), the 70 kDa unpurified GFAP^+1^ band showed co-localization with NF-L (B, up, yellow band). This was gone with the purified antibody, which only stained one GFAP^+1^ band at 50 kDa (B, pur, green band). With the pre-immune serum of the GFAP^+1^ immunized rabbit, no staining was detected (B, pi). The original unpurified GFAP^+1^ antibody stained both neurons and astrocytes in the hippocampus of an AD brain (C, NBB 93-040). These GFAP^+1^ (green) neurons often co-localized with NF-L (red) (D, NBB 01-125), but the astrocytes did not (E, NBB 01-119). The purified GFAP^+1^ antibody only stained a subpopulation of astrocytes (F, NBB 95-102). Scale bars represent 100 µm (C, F), 50 µm (D–E).

A western blot on human spinal cord filaments stained for GFAP^+1^ resulted in two bands; one band migrating at approximately 70 kDa and one band at approximately 50 kDa (Fig. 2A, unpur). After affinity purification of the antibody only the 50 kDa band remained visible (Fig. 1A, pur), which is the expected height of GFAP^+1^ protein as we confirmed by recombinant protein (not shown). Two bands at the same heights were also visible on the Coomassie protein gel (not shown) and these bands were isolated and analyzed by Maldi analysis. Peptide Mass Fingerprinting analysis of the band migrating at approximately 50 kDa revealed it to be human GFAP (MOWSE score: 303 with 28 matching peptides). The band migrating at approximately 70 kDa contained human Neurofilament Triplet L Protein (NF-L) (MOWSE score: 218 with 22 matching peptides). No peptides possibly derived from human GFAP could be detected in the upper band. Accordingly, co-localization of the unpurified GFAP^+1^ antibody with human NF-L was investigated on a western blot with human spinal cord filaments. The NF-L antibody stained a band at approximately 70 kDa, which co-localized with the band stained by unpurified GFAP^+1^. With the purified GFAP^+1^ antibody only the 50 kDa band was detected, whereas no bands were detected with the pre-immune serum of the GFAP^+1^-immunized rabbit (Fig. 2B). Next, immunohistochemistry with the purified GFAP^+1^ antibody was performed on human hippocampus slices of Alzheimer donors and compared to the unpurified antibody. Both neurons and astrocytes were stained with the unpurified GFAP^+1^ antibody (Fig. 2C). Some of these GFAP^+1^ positive neurons clearly co-localized with NF-L (Fig. 2D), but the astrocytes never co-expressed NF-L (Fig. 2E). In agreement with the western blot results, only astrocytes were detected in the sections stained with the purified antibody (Fig. 2F). Additionally, a western blot on homogenates of AD and control brains was performed, but no protein bands were detected after staining with the purified GFAP^+1^ antibody, unlike staining with a pan-GFAP antibody (not shown). Most likely the number of GFAP^+1^ expressing cells in the brain is too low to detect the level of the GFAP^+1^ protein in whole homogenates.

**Figure 3:**
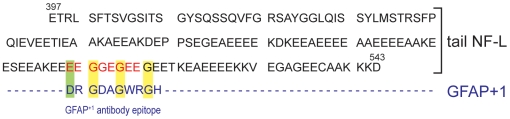
Sequence homology between neurofilament-L and the GFAP^+1^ antibody epitope. Some homology (green and yellow highlights) was found between the tail domain of NF-L (red letters) and the GFAP^+1^ antibody epitope (blue letters).

Comparing the sequences of NF-L and GFAP^+1^ peptide revealed some homology in the tail domain of NF-L (Fig. 3), which might explain the cross-reactivity that occurred. Because not all GFAP^+1^ positive neurons expressed NF-L and vice versa, we hypothesize that a specific conformational change of NF-L in tangle-like structures, induced by the AD pathogenesis, exposed the epitope in NF-L that cross-reacted with the unpurified GFAP^+1^ antiserum.

### Purified GFAP^+1^ Identifies a Specific Subpopulation of Astrocytes in the Adult Human CNS

**Figure 4:**
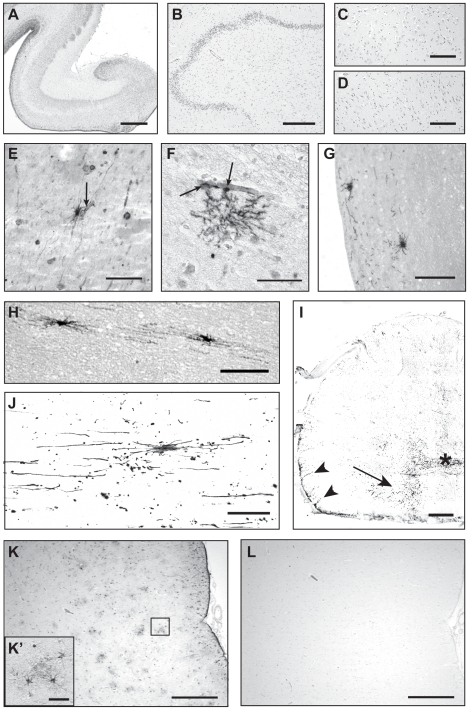
GFAP^+1^ expressing astrocytes in different areas of the human CNS. No GFAP^+1^ expression is detected in the developing hippocampus of a 16 week old human fetus (A), or in the hippocampus of a 39 year old healthy female, neither in the dentate gyrus/hilus (B) nor in CA1 (C) or CA3 (D) regions. The purified GFAP^+1^ antibody clearly stained a subpopulation of astrocytes throughout the brain, e.g. in the caudate/putamen (E–F), along the subventricular zone of the lateral ventricle beneath the cingulate gyrus (G) and in the most posterior part of the lateral ventricle (H). Often cells were found contacting blood vessels (E–F, arrows). A relatively large number of GFAP^+1^ expressing cells was detected in the human spinal cord (I–J). Most expression was found near the meninges (arrowheads), in the anterior horn (arrow) and in the grey commissure around the central canal (asterix) as shown in half of a transverse section (I). A GFAP^+1^ expressing cell and surrounding processes are shown in more detail in a longitudinal section (J). Pan-GFAP staining reveals many reactive astrocytes in the hippocampus (K), which clearly show intense GFAP expression and hypertrophic cell bodies in a magnified image of the squared area (K'). GFAP^+1^ staining in the same area of an adjacent section shows no staining at all (L), indicating GFAP^+1^ does not generally mark reactive astrocytes. NBB 01-021 (E), NBB 02-013 (F), NBB 07-006 (G-H), AMC 06/9 (I–J), NBB 05-083 (K–L). Scale bars represent 500 µm (A–B, K–L), 200 µm (C–D),100 µm (E, G–H, J, 50 µm (B, K'), 1 mm (I).

During development (Fig. 4A) and in the hippocampus of healthy young adults (<50 years), no GFAP^+1^ expression was detected with the purified GFAP^+1^ antibody either in the dentate gyrus/hilus region (Fig. 4B) or in the CA areas (Fig. 4C–D). Immunostaining with the purified GFAP^+1^ antibody revealed conspicuous astrocytes in numerous areas of the CNS in older control donors (>50 years) or donors with neurodegenerative diseases. In addition to the hippocampus of Alzheimer (n = 6) and control brains (n = 6), GFAP^+1^ positive astrocytes were found in the striatum (caudate/putamen) of control and Parkinson brains (Fig. 4E–F). Furthermore, GFAP^+1^ positive astrocytes were found in different areas aligning the subventricular zone, e.g. beneath the cingulated gyrus and in the most posterior part of the lateral ventricle. In these areas cells were found both in the subventricular zone (Fig. 4G) as in more superficial layers (Fig. 4H). Fluorescent immunostaining of transverse (Fig. 4I) and longitudinal (Fig. 4J) sections of the human spinal cord showed a relatively large number of GFAP^+1^ positive astrocytes. Most intense staining was found near the meninges (Fig. 4I, arrowheads), in the anterior horn (Fig. 4I, arrow) and in the grey commisure around the central canal (Fig. 4I, asterix). Since the investigated brain areas are known to be full of GFAP positive astrocytes throughout the area, clearly GFAP^+1^ is only expressed in a specific subpopulation of astrocytes. Besides GFAP^+1^ expression, this subpopulation of astrocytes is characterized by long processes which often contact blood vessels (Fig. 4E–F, arrows) and their morphology resembles reactive astrocytes in that sense that GFAP^+1^ astrocytes are larger in size compared to normal resting astrocytes, however the extensions of the GFAP^+1^ astrocytes are smaller in diameter. Nevertheless, in areas that are full of reactive astrocytes, as indicated by immunostaining with a pan-GFAP antibody (Fig. 4K–K'), often no GFAP^+1^ staining can be detected (L).

## Discussion

In this study we showed that GFAP^+1^ is not expressed in neurons as was previously reported [5]. The neuronal immunoreactivity appeared to be due to cross-reactivity of the antibody with NF-L, which we uncovered by peptide mass fingerprinting. However, GFAP^+1^ is expressed in a specific subpopulation of astrocytes throughout the brain as revealed by the affinity purified GFAP^+1^ antibody.

How immunization with the GFAP^+1^ peptide exactly led to the production of NF-L recognizing antibodies is not known. The homology of the GFAP^+1^ peptide with 4 amino acids in the tail domain of NF-L (Fig. 3) could be an explanation. Furthermore, the issue why only neurons in AD were labeled by the unpurified GFAP^+1^ antibody for NF-L remains unexplained. Perhaps, the antiserum only detects the monomeric form of NF-L, as was shown for the 70 kDa band on the western blot (Fig. 2B). This form has been shown to accumulate in hippocampal neurons of AD brains [9]. Most neurons with NF-L accumulation resembled intact neurons, whereas only little NF-L immunoreactivity was found in tangles. This could also explain why many GFAP^+1^ positive neurons were found to have visible nuclei [5].

The affinity purified GFAP^+1^ antibody revealed a strong and specific immunolabeling of a subpopulation of astrocytes in the hippocampus of Alzheimer donors, and did not label neurons at all. However, neuron-like structures were still labeled by several other GFAP antibodies. In an earlier study we have described that three other pan-GFAP antibodies clearly labeled neuron-like structures [5], but we show in this study that these were not neurofilament positive. These GFAP immunopositive neuron-like structures were also distinct from the neurons that were labeled with the unpurified GFAP^+1^ antiserum. The neuron-like structures were less intensely labeled with the different GFAP antibodies used in our earlier study and with the novel GFAP antibodies we used in this study, that are directed against the C-terminus or N-terminus of human GFAPα. We suggest, as others have done before, that the GFAP positive neuron-like structures are ghost tangles, which are neurofibrillary tangles (NFTs) emerging into extracellular space that are associated with prominent astrocytic invasion including glial filaments [10], [11]. In a study by Ikeda *et al*., these ghost tangles were shown to be GFAP positive and the GFAP staining that they show strongly resembles the GFAP positive neuron-like structures that we showed in our study [10].

Interestingly, the specific GFAP^+1^ immunolabeling in astrocytes remained present after affinity purification. This expression pattern did not get much attention before since GFAP expression in astrocytes is common, unlike neuronal expression. However, the purified GFAP^+1^ antibody intensely stained a small number of large astrocytes with several long processes often directed towards blood vessels. This subpopulation of astrocytes was found in the hippocampus, the striatum, the spinal cord and in several areas aligning the subventricular zone in brains of elderly non-demented controls, Alzheimer and Parkinson patients. GFAP^+1^ expression was not present during brain development or in brains of young controls. This indicates that GFAP^+1^, in addition to the previously described GFAP isoform GFAPδ in subpial and subventricular zone astrocytes [12], marks a specific subpopulation of astrocytes. GFAP^+1^ immunostaining of gliotic brain areas revealed that this GFAP^+1^ expressing subpopulation is distinct from the population of reactive astrocytes. More elaborate characterization of these astrocytes and the possible role of the GFAP^+1^ protein therein will be further investigated. In light of the increasing interest in the function of astrocytes and the subtypes of astrocytes [13], [14], our antibodies might be of value to identify specific subtypes. Furthermore, the different GFAP-IF networks might be instrumental in revealing the diverse functions of specific astrocyte subtypes.
